# The sRNA RyhB Regulates the Synthesis of the *Escherichia coli* Methionine Sulfoxide Reductase MsrB but Not MsrA

**DOI:** 10.1371/journal.pone.0063647

**Published:** 2013-05-09

**Authors:** Julia Bos, Yohann Duverger, Benoît Thouvenot, Claude Chiaruttini, Christiane Branlant, Mathias Springer, Bruno Charpentier, Frédéric Barras

**Affiliations:** 1 Laboratoire de Chimie Bactérienne, Institut de Microbiologie de la Méditerranée, Centre National de la Recherche Scientifique-Aix Marseille Université, Unité Mixte de Recherche, Marseille, France; 2 Centre National de la Recherche Scientifique-Université de Lorraine, Unité Mixte de Recherche, Biopôle de l’Université de Lorraine, Campus Biologie Santé, Vandœuvre-lès-Nancy, France; 3 Unité Propre de Recherche du Centre National de la Recherche Scientifique, Université Denis Diderot, Paris VII, Institut de Biologie Physico-chimique, Paris, France; CNRS UPR9043, France

## Abstract

Controlling iron homeostasis is crucial for all aerobically grown living cells that are exposed to oxidative damage by reactive oxygen species (ROS), as free iron increases the production of ROS. Methionine sulfoxide reductases (Msr) are key enzymes in repairing ROS-mediated damage to proteins, as they reduce oxidized methionine (MetSO) residues to methionine. *E. coli* synthesizes two Msr, A and B, which exhibit substrate diastereospecificity. The bacterial iron-responsive small RNA (sRNA) RyhB controls iron metabolism by modulating intracellular iron usage. We show in this paper that RyhB is a direct regulator of the *msrB* gene that encodes the MsrB enzyme. RyhB down-regulates *msrB* transcripts along with Hfq and RNaseE proteins since mutations in the *ryhB*, *fur*, *hfq*, or RNaseE-encoded genes resulted in iron-insensitive expression of *msrB*. Our results show that RyhB binds to two sequences within the short 5′UTR of *msrB* mRNA as identified by reverse transcriptase and RNase and lead (II) protection assays. Toeprinting analysis shows that RyhB pairing to *msrB* mRNA prevents efficient ribosome binding and thereby inhibits translation initiation. *In vivo* site directed-mutagenesis experiments in the *msrB* 5′UTR region indicate that both RyhB-pairing sites are required to decrease *msrB* expression. Thus, this study suggests a novel mechanism of translational regulation where a same sRNA can basepair to two different locations within the same mRNA species. In contrast, expression of *msrA* is not influenced by changes in iron levels.

## Introduction

Reactive oxygen species (ROS) can damage most macromolecules, proteins, nucleic acids and lipids [1]. Within proteins, sulfur-containing amino acids, cysteine and methionine (Met) exhibit high sensitivity [2]. In particular, methionine oxidation yields methionine sulfoxide (Met-SO) and eventually methionine sulfone. Like any other oxidative modification, methionine oxidation can have deleterious consequences as it can be accompanied by protein carbonylation, protein aggregation and/or degradation [3], [4], [5]. However, the oxidation of Met to Met-SO can be reversed by the action of methionine sulfoxide reductases (Msr) [6], [7], [8], [9], [10]. Such an ability to reverse methionine oxidation has led credence to the idea that the surface-exposed Met residues act as scavengers for ROS and that reduction by Msr enables proteins to recover activity [11].

Msr proteins are highly conserved among most living organisms [12], [13]. There are basically two types, referred to as MsrA and B, which act on two different diastereoisomers, Met-S-SO and Met-R-SO, respectively [8], [10], [14]. Lack of functional MsrA and/or B have been reported to cause abnormal cellular function in a wide range of organisms from bacteria to humans, including yeast, mice, flies and plants [7], [15], [16], [17], [18], [19], [20], [21]. Likewise neurological disorders, shortened life span, and various oxidative stress-related defects have been reported in eukaryotes having reduced Msr activity. As a general trend, bacteria lacking Msr exhibit hypersensitivity to ROS and those that are pathogens have a reduced ability to infect their host [22], [23], [24], [25], [26], [27], [28], [29].

Small non-coding RNAs are believed to play a major role in genome expression both in prokaryotes and eukaryotes. The *E. coli* iron-responsive sRNA RyhB that controls iron metabolism is one of the best studied. Early microarray approaches suggested that RyhB controls directly the expression of about 50 genes in response to iron limitation [30]. RyhB expression is itself negatively regulated by the iron-sensing Ferric uptake regulator (Fur) [31]. Hence, when iron becomes limiting, Fur repression is alleviated, RyhB synthesis is induced and expression of its targets inhibited. Genes targeted by RyhB encode iron-storage and iron-using proteins and it was proposed that RyhB enables iron sparing for central metabolism and for essential iron-binding proteins when iron concentration becomes scarce [30], [31], [32]. Therefore, synthesis of a non-essential-iron-using protein would be decreased and the cell would rather employ an iron-non-using functional homolog. An illustration of this model is provided by the SodA and SodB superoxide dismutases. The synthesis of the iron superoxide dismutase SodB is decreased by RyhB under iron limitation, whereas the synthesis of SodA, a Mn-superoxide dismutase is not [30], [33]. These iron-responsive regulators, RyhB and Fur also play a crucial role to reduce the response to oxidative stress, as free iron is susceptible to react with ROS such as superoxide anions and to increase ROS production via the Fenton and Haber Weiss reactions [34]. RyhB regulates gene expression by basepairing within or near the translation initiation region of mRNAs. Two mechanisms of action have been proposed for RyhB to explain its role as a gene expression regulator. In a first mechanism, the pairing of RyhB to its target mRNA interferes either positively or negatively with the 30S ribosome subunit binding and thereby translation [31], [35], [36]. Alternatively, the pairing of the sRNA RyhB to the target mRNA can initiate mRNA degradation even in the absence of translation on the mRNA target [37].

Early microarray analysis suggested that RyhB levels influence the expression of *msrB* but not that of *msrA* [30]. In addition, MsrB has been biochemically characterized as an iron binding protein [38]. Therefore we wished to test the functional and direct inactivation of the *E. coli msrB* mRNA by RyhB *in vitro* and *in vivo*, and whether the cell switches to *msrA* under iron limitation. We provide evidence that RyhB directly pairs with the 5′UTR region of *msrB* at two distinct sites and does repress *msrB* expression by competing with the ribosome. Secondly, we showed that *msrA* expression is not sensitive to changes in iron availability. Thus, in addition to identifying a new target for the sRNA RyhB and pointing out a novel mechanism of translational regulation, this study indicates that in *E. coli* MsrB is dispensable when iron supply is low but MsrA is retained to repair even limited methionine oxidation.

## Materials and Methods

### Media and growth conditions

Derivatives of *E. coli* MG1655 strain were used in all experiments and were cultivated under aerobic conditions at 37°C in Luria Bertani (LB) medium unless stated otherwise. Plasmids were maintained with ampicillin used at a final concentration of 100 μg/ml.

### Plasmid construction

Transcriptional and/or translational fusions of *msrB* and *msrA* genes to *lacZ* reporter gene were constructed by inserting *msrB* fragment (from −336 to +360 relative to *msrB* start codon) or *msrA* fragment (from −416 to +132 relative to *msrA* start codon) into plasmids pRS415 (transcriptional fusion) and pRS414 (translational fusion)[39]. For information, pRS415 contains the *lacZ* ORF including the *lacZ* translation initiation region (TIR) whereas pRS414 contains the *lacZ* ORF without the *lacZ* TIR (‘*lacZ*). *msrB and msrA* genes were amplified from MG1655 chromosomal DNA with the oligonucleotides B01E/B05B and A01E/A04B (see Table 1) respectively. The resulting products were digested with *Eco*RI and *Bam*HI and ligated into *Eco*RI/*Bam*HI-digested pRS415 and pRS414 plasmids, to generate the transcriptional and translational fusions to *lacZ* gene. The *msrB’-lacZ*, *msrB’-‘lacZ* and *msrA’-‘lacZ* constructs were further engineered onto λRS45 phage and integrated into the attachment site (*att* site) of IBPC5321 strain as previously described [39], generating strains JB43 (transcriptional *msrB’-lacZ*), JB44 (translational *msrB’-‘lacZ*) and JB56 (translational *msrA’-‘lacZ*). Stable lysogens were screened for single insertion of recombinant by PCR [40] and sequenced using LacZinv primer (see Table 1). We next introduced the following Δ*fur ::kan* and Δ*ryhB ::cat* deletions by P1 transduction in JB44 generating strains JB46 and JB50 respectively and in JB56 to generate YD100.

**Table 1:** List of oligonucleotides used in this study.

Name	Sequence
LacZinv	ATCGGTGCGGGCCTCTTC
BO1E	CGCGAATTCCCACCAGCTATTTGTTAGTG
BO5B	CGCGGATCCGGGTTAACACAATAACGTTCGCCCGTTGG
BO5N	GGGCATATGTCAACCGTTGATTTCTTCGCCGTT
AO1E	CGCGAATTCCCTTTCAGACCTTCGCGGATG
AO4B	CGCGGATCCGTGCCGATCAGGCCTACGCAG
5S1	GCGGTCTGATAAAACAGAATTTGCC
5S2	GCCTGGCAGTTCCCTACTCTCGCAT
BT7	TAATACGACTCACTATAGGGCACCAGCTATTTGTTAGTGA
B1	CCTTCGGCAGAAGAACTG
B4	GCCCGTTGGCTGCGGCCCGTCGGGGAAGACATG
RT7	TAATACGACTCACTATAGGGGCGATCAGGAAGACCCTCGC
Reco	GCGGAATTCCGCGATCAGGAAGACC
Rbam	GCGGGATCCAAAAAGCCAGCACCCGGC
PUCVERI	GGGCATTAGGCACCCCAGGCTTT
BM1for	GAATCAGTCACGTAATACAAGCACGATTAAAGTGAGATG
BM1rev	CATCTCACTTTAATCGTGCTTGTATTACGTGACTGATTC
BM2afor	ACGATTAAAGTGAGATGTGACTCAATGGCTAATAAACCTTCGG
BM2arev	CCGAAGGTTTATTAGCCATTGAGTCACATCTCACTTTAATCGT
BM2bfor	ACGATTAAAGTGAGATGTGAGATAATGGCTAATAAACCTTCGG
BM2brev	CCGAAGGTTTATTAGCCATTATCTCACATCTCACTTTAATCGT
437	GATTCTGCGTCACGCGTGACGC
1429	GGCGGTTCTGTCCCATGATTCTGCGTCACGCGTGACGC
3261	GACTCACTATAGGGGTCACGTAATGTGAGCACGATTAAAG
BHA	GACTCTCACGTGCATATGTCATTAAGCGTAGTCTGGGACGTCGTATGGGTAACCGTTGATTTCTTCGCC

The construct pUC-MsrB was generated by PCR using chromosomal DNA from MG1655 wild type strain as a template and primers B01E and B05N (see Table 1). The resulting product was digested wih *Eco*RI and *Nde*I and cloned into the corresponding sites of pUC18. The construct was sequenced using the primer pUCVERI and transformed into MG1655 wild type strain. The construct pUC-MsrB-HA has been generated using the same protocol to that of pUC-MsrB construct. The HA tag was added to the 3’ end of *msrB* using the reverse oligonucleotide BHA (see Table 1). IPTG (0.5 mM) was added to the culture to induce the synthesis of MsrB and MsrB-HA. Strains used in this study are listed in Table 2.

**Table 2:** List of bacterial strains and plasmids used in this study.

Strains	Relevant genotype	Source or reference
MG1655	wild type E. coli K12	Lab collection
IBPC5321	*thi-1,argE3, lacX74, mlt-li, xyl-5, tsx-29, rpsL,argG6, his4*	[56]
JB43	IBPC5321 *msrB’-lacZ*	This study
JB44	IBPC5321 *msrB’-‘lacZ*	This study
JB46	IBPC5321 *msrB’-‘lacZ Δfur::kan*	This study
JB50	IBPC5321 *msrB’-‘lacZ ΔryhB::cat*	This study
JB55	IBPC5321 *msrA’-lacZ*	This study
JB56	IBPC5321 *msrA’-‘lacZ*	This study
YD100	IBPC5321 *msrA’-‘lacZ ΔryhB::cat Δfur::kan*	This study
JB64	IBPC5321 *msrA’-‘lacZ ΔryhB::cat*	This study
JB61	IBPC5321 *msrA’-‘lacZ Δfur::kan*	This study
LCB195	MG1655 *Δfur::kan*	Lab collection
JB99	MG1655 *ΔryhB::cat* (MG1655*P1 EM1238)	This study
JB91	MG1655 *rne701-flag-cat* (MG1655* P1 TM528)	This study
SMG505	MG1655 *msrB::kan*	Lab collection
EM1238	EM1055 *ΔryhB::cat*	[31]
TM528	W3110 *mlc rne701-flag-cat*	[57]

Plasmids	Description	Source or reference
pRS414	contains ‘*lacZ* ORF (deleted TIR)	[39]
pRS415	contains full *lacZ* ORF	[39]
pRS414-MsrB’-‘LacZ	*msrB-336/+360* fused to *lacZ* ORF	This study
pRS415-MsrB’-LacZ	*msrB-336/+360* fused to *lacZ* ORF	This study
pRS414-MsrA’-LacZ	*msrA-416+132* fused to ‘*lacZ* ORF	This study
pUC-MsrB	pUC18 carrying wild type *msrB*	This study
pUC-MsrBmut1a	pUC18, carrying mutated *msrBA9G,G10A,C11U*	This study
pUC-MsrBmut1b	pUC18, carrying mutated *msrBG6A,U7C,G8A*	This study
pUC-MsrBmut2a	pUC18, carrying mutated *msrBG32C,C33U,A34C*	This study
pUC-MsrBmut2b	pUC18, carrying mutated *msrBC33A,A34T*	This study
pUC-MsrB-HA	pUC18 carrying HA tagged-*msrB* (wt)	This study
pUC-MsrBmut2aHA	pUC18 carrying HA tagged-*msrB* (*mut2a*)	This study
pUC-MsrBmut2bHA	pUC18 carrying HA tagged-*msrB* (*mut2b*)	This study
pUC-RyhB	pUC19 carrying wild type RyhB	B. Charpentier (UHPNancy)

### Site-directed mutagenesis

Site-directed mutagenesis was carried out by PCR using pUC-MsrB or pUC-MsrB-HA as DNA template. Mutations *mut1*, *mut2a*, and *mut2b* are introduced into the 5’UTR region of *msrB* with the use of primers Bm1for-Bm1rev, Bm2afor-Bm2arev, Bm2bfor-Bm2brev respectively, containing the suitable nucleotide mismatches (see Table 1). The amplified plasmid products were then digested with *Dpn*I restriction enzyme for 2 hours at 37°C to select for mutation-containing synthesized DNA and 1 μl aliquot was transformed into DH5 alpha competent cells. Transformation reactions were plated on LB containing ampicillin. Five colonies were selected and the positive clones were screened by digestion and sequenced. Plasmids carrying wild type or mutant alleles were introduced into Δ*msrB* strain (SMG505). Transformants were used for Northern blot and Western blot experiments.

### Western blotting

Bacteria cells carrying pUC-MsrB-HA, pUC-MsrB_mut2a_-HA and pUC-MsrB_mut2b_-HA were cultivated in LB medium to exponential phase. Iron chelator (2–2′ dipyridyl) was added for 30 min and 50 ml sample aliquots were collected. French Press lysates were spun down for 30 min at 13500 rpm. Supernatants were collected and equal amounts of cellular extracts were western-blotted. To detect MsrB-HA protein, the membrane was probed with a mouse anti-HA primary antibody, and with a goat anti mouse IgG alexa fluor 680 as secondary antibody and scanned by using an Odyssey IR imaging scanner. Band intensity was analyzed by using Image J software.

### DNA probe labeling

Radiolabeled probes used in the Northern blots were generated from PCR products. *msrB*, RyhB and 5S PCR products were generated using the pair of oligonucleotides B1–B4, REco-Rbam and 5S1-5S2 respectively (see Table 1). After gel extraction and purification, the PCR products were then used as a template for [α-^32^P]dCTP-labeling reaction (Ready-to-go DNA labeling kit, Amersham). Unincorporated nucleotides were removed by using a G-50 resin minicolumn. For primer extension assays and toeprint assays, the 5′ end of oligonucleotides 437 and 1451 respectively, was labeled with [γ-^32^P]ATP (50 µCi) using T4 polynucleotide kinase (10 U).

### RNA isolation and Northern blot analysis

Total RNA was isolated from cells at the indicated times using the SV total RNA isolation system kit as described by the manufacturer (Promega). For Northern blot analysis, total RNA (10 µg) was separated on a 1.2% TBE-agarose gel and transferred to a Hybond N+ nylon membrane. Membranes were UV-crosslinked and hybridized for 4 hours at 58°C with the suitable radiolabeled probe. Membranes were washed three times in 0.05%SDS/2X SSC buffer and then twice in 0.1%SDS/0.1X SSC buffer before being scanned by using a phophorimager (Molecular Dynamics). Band intensity was analyzed by using Image J software.

### Primer extension assays

Hybridization reaction between of 5′-end labeled oligonucleotide 437 (4 ng) and purified *msrB* RNA transcript (100 ng) was performed in a final volume of 2.5 µl, in the 1X binding buffer (Tris HCl 10 mM, pH8, MgCl_2_ 10 mM, NaCl 50 mM, KCl 50 mM) for 10 min at 65°C. RyhB sRNA (5 µg) was added to the reaction mix (7.5 µl) for 10 min at 37°C. The reverse transcription reaction was performed for 30 min at 42°C by adding 5 µl of the AMV RT enzyme and dNTPs to the reaction mix. The resulting cDNA reaction mix was loaded onto a 6% acrylamide/bisacrylamide denaturing gel along with the products of dideoxy-sequencing reactions obtained with the same labeled primer. Signal intensities were evaluated using a phosphorimager (Molecular Dynamics).

### Toeprinting assays

Toeprint experiments were carried out using purified 30S ribosomal subunits (isolated from D10 *E. coli* strain as described in [41]), tRNAfMet and AMV reverse transcriptase to detect the formation of ternary initiation complexes on the long *msrB* transcript (450 nt). In a typical experiment, the *msrB* RNA transcript (50 fmol) was mixed to a fourfold molar excess of 5′-labeled 437 oligonucleotide primer (complementary to positions +60 to +73 of *msrB*). Toeprint reactions were performed as described in [42]. The toeprint band intensity was analyzed by using Image J software and normalized to that of full-length transcript. Data are represented as means ±S.E. from two independent experiments.

### Half-life determination

Overnight cultures were subcultured in LB medium (200 ml) at 37°C to an O.D._600_ of 0.3. Each culture was then divided in 2 and one culture was treated with 2–2′ dipyridyl (250 µM) for 15 min at 37°C. Samples of 5 ml started to be collected at different times (0, 2, 4, 6, 8, 10, 12, 15 and 30 min) right after the addition of Rifampicin (0.3 mg/ml) and were immediately frozen in liquid nitrogen. Total RNA was extracted from each sample and 10 µg of total RNA were subjected to a Northern blot analysis using the specific *msrB* and 5S probes. Signal intensities were evaluated using a phosphorimager (Fuji FLA 5100) and quantitated by using Image J software. *msrB* signals were normalized to that of 5S RNA. Data are represented as means ±S.E. from three independent experiments.

### Beta-galactosidase assays

Cultures were grown to exponential phase (O.D.600≈0.4) in LB medium supplemented or not with 250 μM 2,2′ dipyridyl iron chelator. The β-Galactosidase assays were carried out as described (Miller, 1972). The results are reported in Table 3.

**Table 3:** Expression of *msrA-lacZ* and *msrB-lacZ* fusions in wild type, *ryhB* deletion or *fur* deletion strains grown to exponential phase (O.D._600_≈0.4) in LB rich medium either in the presence or in the absence of iron chelator (2,2′dip, 250 µM).

Type of fusion	Strains	LB	LB +2,2′dip	Fold expression ±2,2′dip
Transcriptional	*msrB’-lacZ* (JB43)	1655±35	1502±30	0.9x
Translational	*msrB’-‘lacZ* (JB44)	680±20	190±8	0.3x
Translational	*msrB’-‘lacZ ryhB ::cat* (JB50)	688±16	702±8	1.0x
Translational	*msrB’-‘lacZ fur ::kan* (JB46)	181±6	172±9	1.0x
Transcriptional	*msrA’-lacZ* (JB55)	105±32	148±35	1.4x
Translational	*msrA’-‘lacZ* (JB56)	58±14	79±4	1.4x
Translational	*msrA’-‘lacZ ryhB ::cat* (JB64)	53±6	93±13	1.7x
Translational	*msrA’-‘lacZ fur ::kan* (JB61)	53±5	60±10	1.1x
Translational	*msrA’-‘lacZ ryhB ::cat fur ::kan* (YD100)	34±5	47±14	1.4x

The level of β-galactosidase expressed from the fusions is given in Miller units. Each value is the average of four independent experiments with three measurements each. Fold expression in iron starvation is indicated. Standard error is indicated.

### In vitro transcription of msrB and RyhB

In vitro transcription of the *msrB* mRNA and the RyhB sRNA was performed using 200 units of T7 polymerase in T7 transcription buffer (Ambion), the pUC-RyhB and pUC-MsrB vectors (0.5 μg) as a DNA template respectively. After 2 h of incubation at 37°C, the mixture was treated with RNase-free DNase I for 30 min at 37°C. RNA transcripts were extracted by phenol/chloroform followed by ethanol-precipitation and were resuspended in RNase-free water. Concentrations of *msrB* and RyhB transcripts were determined by using a WPA BIOWAVE II spectrophotometer. Dephosphorylated RNA transcripts were 5′ end-labeled with [γ-^32^P]ATP using T4 polynucleotide kinase for 30 min at 37°C. Radiolabeled RNAs were purified onto a denaturing (8 M urea) polyacrylamide gel and eluted overnight in elution buffer (10 mM Tris-HCl pH 7.5, 300 mM NaCl, 1 mM EDTA, 1% SDS) followed by ethanol precipitation.

### RNases and Lead (II) footprinting

^32^P-5′ end-labelled *msrB* RNA (∼ 50 fmol) was incubated in buffer D [150 mM KCl, 1.5 mM MgCl_2_, 0.2 mM EDTA and 20 mM HEPES (pH 7.9)]. For ribonuclease cleavage, reactions were initiated by the addition of 10^−5^ U/ml of RNase V1 or 5×10^−2^ U/ml of RNase T2, and incubated for 5 min at room temperature. The enzymatic reactions were stopped by extraction with phenol/chloroform and precipitation by ethanol in presence of sodium acetate (0.3 M) and 2 µg tRNAs.

For lead (II) footprinting, the reactions were initiated by the addition of 12 mM Pb(II) acetate. After 5 min incubation at room temperature, reactions were stopped by adding EDTA (0.05 M) before ethanol precipitation followed by extraction with phenol/chloroform and a second precipitation by ethanol in the presence of sodium acetate (0.3 M) and 2 µg tRNAs. As sequence markers, RNA alkaline hydrolysis ladders (cleavage after each nucleotide) were generated by incubating in 1 M sodium carbonate at pH9 for 3 min at 96°C. RNase T1 ladders (cleavage after each guanosine) were generated by incubating the RNA in 1 M sodium hydroxide citrate for 5 min at 65°C, 2 µg tRNAs then by adding 1U RNase T1 for 10 min at 65°C. For both enzymatic and chemical probing reactions, the treated RNA samples were then ethanol-precipitated in the presence of 0.3 M sodium acetate and washed with 80% ethanol. RNA pellets were directly resuspended in RNA loading dye [95% (v/v) formamide, 20 mM EDTA, 0.05% (w/v) each bromophenol blue and xylene cyanol]. The cleavage products were separated onto a 10% polyacrylamide 8 M urea-containing gel and visualized by using a Phosphorimager.

## Results

### Expression of the *msrB* gene is repressed under iron starvation

**Figure 1:**
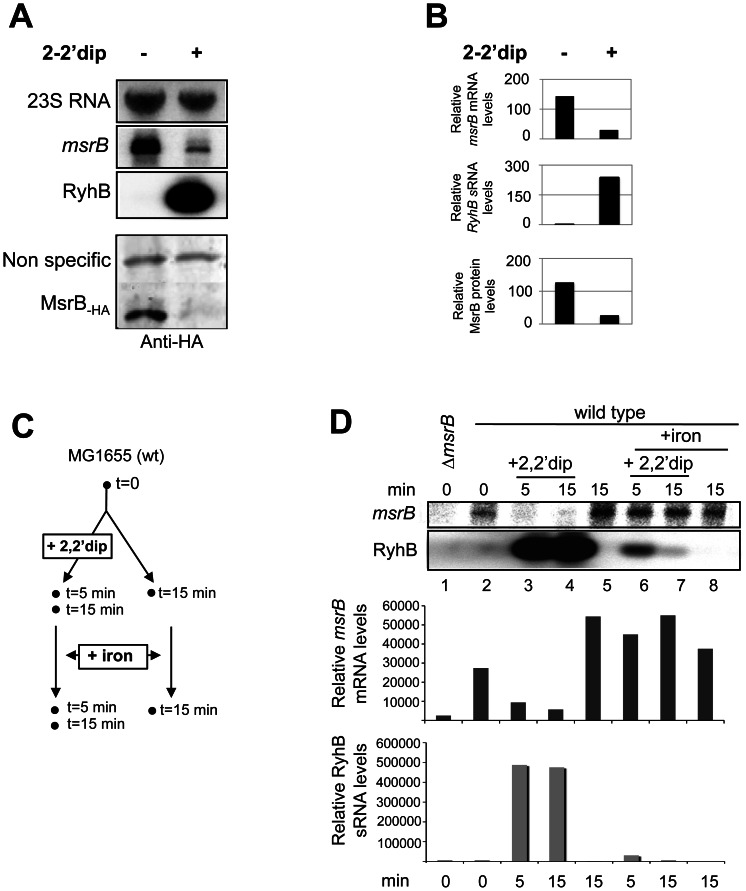
RyhB-dependent down-regulation of *msrB* mRNAs and proteins. (A) Cultures containing a wild type strain of *E. coli* were grown to an O.D._600_ value of 0.5 then 250 μM 2,2′dip was added. After 30 min of incubation with 2,2′dip, total RNA and proteins were extracted in parallel, giving total RNA used for the Northern blots (top panel) and soluble protein fraction used for Western blot (bottom panel). The same membrane was probed successively for *msrB* mRNA, RyhB, and 23S RNA (loading control) (top panel). MsrB proteins were probed with anti-HA antibodies (bottom panel). The radioactive probes used are described in Materials and Methods, and Table 1. (B). Quantification of *msrB* mRNA, RyhB sRNA and MsrB protein levels (arbitrary units) from experiment described in (A). Band intensity was normalized to that of an internal control (23S for both *msrB* and RyhB RNA bands; a non-specific protein recognized by anti-HA antibodies for MsrB-HA protein band). (C). Overview of the experiment described in D. Total RNA was extracted at the indicated times (min). (D). Wild type *E. coli* cells were grown in LB (lanes 1,2,5), LB + 2,2′dip (250 μM) (lanes 3,4). Iron (100 μM) was added after 15 min of growth in LB (lane 8) and after 5 or 15 min of pre-incubation with 2,2′dip (lanes 6–7). Samples were removed at indicated time points, and total RNA was extracted as described in Materials and Methods. Strain SMG505 (Δ*msrB*) was used as a control (lane 1). For determination of RyhB and *msrB* RNA amounts, 10 μg of total RNA samples were loaded onto a denaturating agarose gel. After migration, a Northern blot hybridization was performed with a specific oligoprobe for RyhB and *msrB* respectively. Quantification of *msrB* and RyhB transcript levels (arbitrary units) are shown below Northern blots panels.

Transcriptional and translational fusions between the *msrB* gene and the *lacZ* reporter gene were constructed and inserted at the *att* site in the *E. coli* chromosome. The engineered strains namely JB43 and JB44, respectively, were grown in the presence or absence of 2,2′dipyridyl (2,2′dip), an iron chelator, and ß-galactosidase activity assayed (Table 3). Expression of the translational fusion was repressed by more than three fold in the presence of 2,2′dip whereas expression of the transcriptional fusion remained nearly the same in both conditions. To assess whether the decrease in expression of the translational reporter observed with the 2,2′dip was due to a decrease in both *msrB* mRNA and protein levels, we measured the amount of *msrB* mRNA and MsrB protein within the cell by Northern and Western blot analyses, respectively. Levels of both *msrB* mRNA and MsrB protein showed a 5-fold decrease under iron limitation (Figure 1A–B). The sRNA RyhB is known to specifically down-regulate the expression of numerous genes under iron starvation [30], [31]. Introducing a *ryhB* mutation in JB44 (JB50) abolished 2,2′dip repression, yielding an up constitutive and iron-independent phenotype (Table 3). Expression of RyhB is under Fur repression. Introducing a *fur* mutation in JB44 (JB46) abolished 2,2′dip repression, yielding a down constitutive iron-independent phenotype (Table 3). Taken together, these results show the expression of the *msrB* gene to be regulated by the Fur/RyhB cascade such that *msrB* expression decreases under iron starvation.

### Expression of the sRNA RyhB reduces the *msrB* transcript levels

The sRNA RyhB promotes the degradation of mRNAs encoding Fe-using proteins and the synthesis of RyhB is induced under iron starvation [31], [33]. Therefore, we analysed the levels of both RyhB and *msrB* transcripts in cells that were submitted to sudden iron starvation and subsequent iron replenishment (Figure 1C, 1D). Briefly, cells were grown to mid-exponential phase, exposed to 2,2′dip for 15 minutes and subsequently exposed to excess iron (100 μM FeSO_4_). We then carried out Northern blots with probes specific to *msrB* and RyhB transcripts. Results showed that within 5 minutes after 2,2′dip exposure, *msrB* transcript levels were greatly decreased (Figure 1D lane 3), while RyhB transcript levels immediately increased (Figure 1D lanes 3–4). When cells were supplemented with excess iron, *msrB* transcript levels reached a maximum within 5 minutes while RyhB transcript decreased to undetectable levels within 15 minutes (Figure 1D, lanes 6–7). These observations establish a clear correlation between the presence of RyhB and the absence of *msrB* transcripts, in full agreement with the hypothesis that RyhB induces *msrB* transcript degradation.

**Figure 2:**
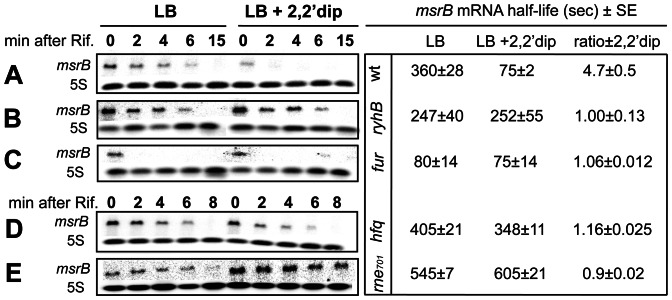
Effects of *ryhB*, *fur*, *rne701* and *hfq* mutations on *msrB* transcript stability. *msrB* transcript stability in wild type (A), *ryhB* mutant (B), *fur* mutant (C), *hfq* mutant (D) and *rne701* mutant (E) strains grown at 37°C to an O.D._600_ of 0.4, was assayed by Northern blot analysis. After 10 min of incubation with 2,2′dip, rifampicin was added. Samples were removed at the indicated time points after rifampicin addition and total RNA was extracted as described in Materials and Methods. For determination of *msrB* mRNA amount, 10 μg of total RNA samples were loaded onto a denaturating agarose gel. After migration, a Northern blot hybridization was performed with a specific oligoprobe for *msrB* and 5S as an internal control. Band intensity of *msrB* transcript was normalized to that of 5S RNA. Half-life (seconds) of *msrB* transcript and the ratio of *msrB* mRNA half-life ±2,2′dip are indicated for the different strains. Standard errors (SE) are shown.

In order to quantify this putative destabilizing effect, we determined the half-life of *msrB* mRNA in the presence and in the absence of RyhB. To measure the half-life, 2,2′dip was added to the culture for 15 minutes, followed by the addition of rifampicin to stop transcription. Total RNA was extracted at different time points and the mRNAs were hybridized with probes specific either to *msrB* or to *5S* RNA as an internal control (Figure 2). In the wild type strain, the half-life of the *msrB* mRNA was 4.7 times shorter in the presence of 2,2′dip. In contrast, in the *ryhB* mutant, the half-life of the *msrB* transcript was the same regardless of the addition of 2,2′dip. Moreover, in a *fur* mutant that synthesizes RyhB at a high-level, the half-life of the *msrB* transcript was the same whether 2,2′dip was added or not and was as short as in 2,2′dip-exposed wild type cells.

Altogether, these observations fit with the model that iron depletion induces RyhB synthesis, which in turn favours the degradation of the *msrB* mRNA.

### The RNA degradosome and the RNA chaperone Hfq are required for the RyhB-induced *msrB* mRNA degradation

Previous characterizations of the mode of action of RyhB showed that RNase E and the RNA degradosome are required in the RyhB-mediated degradation of target mRNAs [33]. Therefore we tested if this also explains the RyhB-mediated decrease in the levels of the *msrB* mRNA. In the *rne701* mutant, a strain lacking a functional RNA degradosome, the half-life of the *msrB* transcript was increased 1.5-fold and 8-fold, as compared with the wild type strain, in the presence and in the absence of 2,2′dip respectively (Figure 2). This indicates that the RNA degradosome is involved in the *msrB* transcript destabilization observed under iron limiting conditions.

The RNA chaperone Hfq is essential for RyhB-mediated regulation of its target genes. Therefore we tested whether Hfq was involved in *msrB* regulation. In an *hfq* mutant, the half-life of the *msrB* transcript was only slightly changed by the presence of 2,2′dip and was increased 4.6-fold in the presence of 2,2′dip as compared with the wild type strain.

Altogether these observations validate that the RyhB/Hfq/Rnase E complex is involved in the *msrB* mRNA decay.

### Identification of two RyhB/*msrB* pairing sites in the 5′ UTR of the *msrB* mRNA

**Figure 3:**
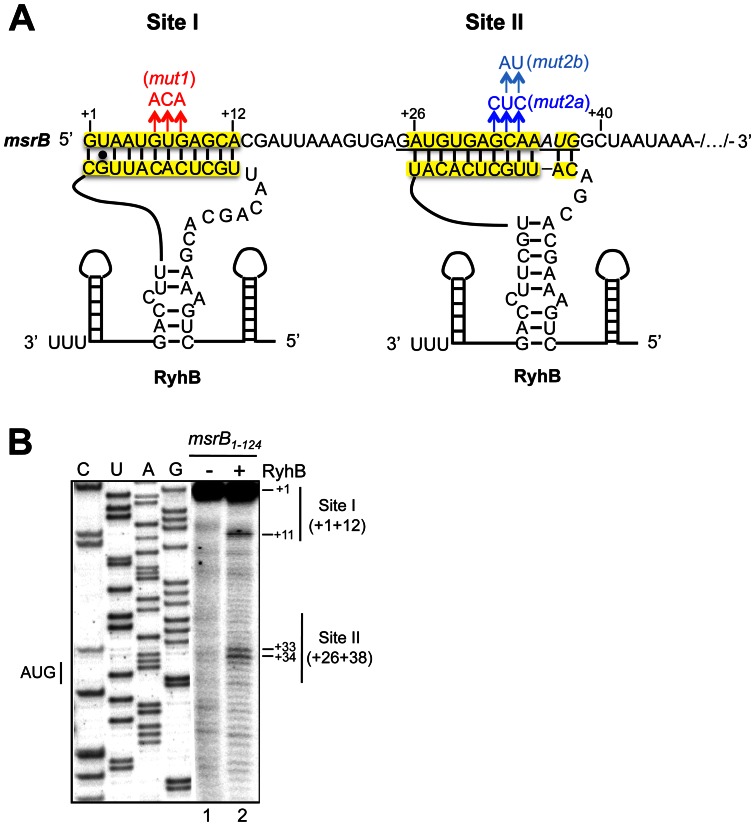
RyhB binds *msrB* mRNA at two sites. Panel (A) shows the predicted interactions between RyhB and the *msrB* sense strand referred as Site I and Site II (in yellow). The predicted ribosome-binding site for *msrB* is underlined. The start codon for *msrB* is shown underlined and in italics. Mutations in Site I and Site II are shown in red and blue respectively. (B) cDNA extension experiments with wild type *msrB*_1–124_ as a template for the reverse transcriptase. Lane 1: extension with no other component added; lane 2: extension with RyhB alone. C, U, A, and G are sequencing lanes obtained using the same radiolabeled primer as in the reverse extension analysis. Reverse transcriptase stops are indicated at positions +11, +33 and +34. Nucleotides involved in Sites I and II are indicated as thin vertical lines. The transcription start of *msrB* is referred to as the position + 1. The numbers on the left indicate sequence positions with respect to the transcription start.

In order to determine whether the RyhB control of *msrB* expression was due to a direct interaction between the two RNA species, the 5′ untranslated region (5′ UTR) of *msrB* mRNA was searched for sequences complementary to sequences in RyhB. Our results revealed two such potential interaction sites (Figure 3A). The first site, which we will refer to as Site I, is located between G1, the first nucleotide to be transcribed, and A12 (Figure 3A). This 12 nucleotide long sequence exhibits 12 pairings with sequence within RyhB stem-loop 2 with a total free energy of −25.4 kcal/mol. The second site, which we will refer to as Site II, is located between A26 and G38 and hence overlaps the Shine-Dalgarno sequence. This 13 nucleotide long sequence exhibits 12 matches with a region located within RyhB loop 2 (Figure 3A). Pairing at Site II between RyhB and *msrB* mRNA is predicted to be less stable than the pairing at Site I with a free energy of −11.7 kcal/mol. Thus, this sequence analysis raised the possibility that *msrB* mRNA possesses two RyhB binding sites. It is remarkable that the two sites contain duplicates of a 9-nucleotide sequence (AUGUGAGCA) and therefore predicted to pair with the same RyhB region, i.e. a sequence of the central stem-loop of RyhB.

### Experimental identification of two RyhB binding sites in the 5′ UTR of *msrB* mRNA

The validity of the predictions reported above was subsequently tested by *in vitro* approaches. First, we asked whether RyhB binding on *msrB* mRNA would block reverse transcriptase (RT) elongation in a primer extension assay (PEA). A shorter version of *msrB* mRNA (124 nt; nt +1 to +124) including the whole 5′ UTR region (35 nucleotides) was transcribed *in vitro* and used as a template for PEA in the presence and in the absence of RyhB (Figure 3B). In the presence of RyhB, two primer extension products were detected that were mapped within Site I (C11) and Site II (C33, A34)(Fig. 3B; lane 2). As a control, we performed a PEA on the *msrB* mRNA alone and observed no specific primer extension product along the *msrB_1–124_* RNA transcript (Figure 3B; lane 1).

**Figure 4:**
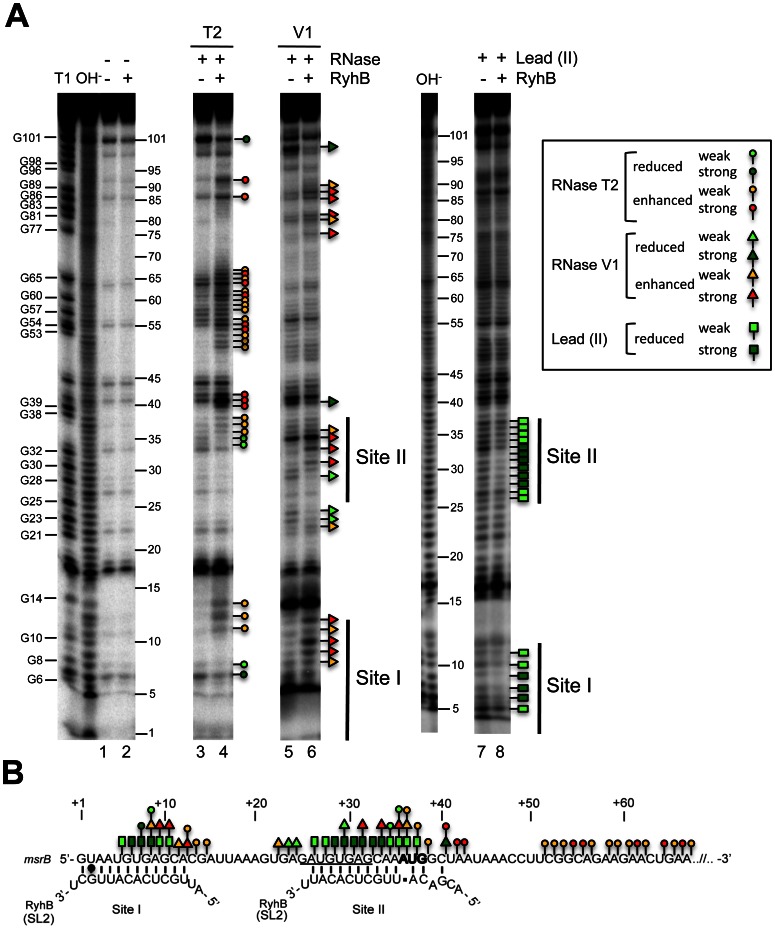
Changes in *msrB* RNA accessibility to enzymatic and chemical probes upon RyhB binding. (A) RNases and lead (II) footprinting: 5′ end-labelled *msrB*_1–124_ transcript was subjected to partial digestion with RNase T2 (lanes 3–4), RNase V_1_ (lanes 5–6) or lead (II) (lanes 7–8) in the presence (+) or in the absence (-) of RyhB sRNA. Lanes 1 and 2 are control lanes of RT extension on *msrB* alone (lane 1) or on *msrB* with RyhB (lane 2). The resulting fragments were then analyzed onto a denaturing sequencing gel. The numbers indicate sequence positions with respect to the transcription start site. Lanes OH^−^ and T1 correspond, respectively, to an alkaline hydrolysis ladder, and an RNase T1 digestion ladder obtained in denaturing conditions. The position of G residues that resulted from RNase T1 hydrolysis is given. Circles, arrowheads, and rectangles indicate, respectively, phosphodiester bonds cleavages by RNase T_2_, RNase V_1_, and lead (II). Products resulting from a strong (red) or a weak (orange) enhancement of the cleavages in presence of RyhB are indicated. Reduced levels of cleavages in presence of RyhB are indicated by dark green (strong) and light green (weak) symbols. RyhB-binding sites (Sites I and II) are shown as thin vertical lines. (B). Summary of the RNases/lead (II) footprints of *msrB*_1–124_ mRNA in the presence of RyhB based on the results obtained in (A). The translation start codon of *msrB* is shown in bold and the Shine Dalgarno sequence is underlined. RyhB Stem Loop 2 (SL2) pairing at Site I and Site II is shown. The same rules as in panel A are utilized for representation of changes in phosphodiester bonds cleavages in presence of RyhB.

Second, we compared patterns of partial digestion of the 5′ UTR of the *msrB* transcript with RNAses in the absence and in the presence of RyhB (Figure 4). When the *msrB* mRNA was digested by RNase T2 (a single strand-specific endoribonuclease with preference for adenosine residues) in the presence of RyhB, cleavages appeared at the 3′ end of Site I (bonds between residues at positions +12 to +14) and Site II (bonds between residues at positions +40 to +42) while some others were strongly reinforced downstream of the Site II (bonds between residues at positions +51 to +67) (Figure 4, lane 3). Conversely, the presence of RyhB afforded protection against RNase T2 cleavages within the binding Sites I and II (bonds between residues at positions +7 and +8, and at positions +34 and +35, Figure 4; lane 4). When RNase V1 (specific for double stranded regions and stacked nucleotides) was used in the presence of RyhB, significant changes in the RNase V1-digestion pattern were observed with new cleavage products in the sequence of the predicted binding Site I (bonds between positions +8 to +11) and Site II (bonds between positions +31/+32, +33/+34 and +35 to +37) (Figure 4; lane 6). Additional cleavages were also observed within the coding region of *msrB* but no potential RyhB binding sites was detected by sequence analysis and no prematured RT extension products were observed (data not shown). Last, digestion of the 5′UTR of *msrB* with lead (II) acetate (specific for single stranded regions) in the presence of RyhB (Figure 4, lane 8) showed that the two regions that refer to Sites I and II were significantly protected from lead (II) digestion (bonds between positions +5 to +11 and +26 to +38, Figure 4B).

Thus, taken together, PEA, RNase/lead (II) probing analyses fully establish that RyhB pairing occurs at two sites, referred to Sites I and II within the short 5′ UTR of the *msrB* mRNA.

### Mutagenesis analysis of the RyhB/*msrB* interaction *in vitro*

**Figure 5:**
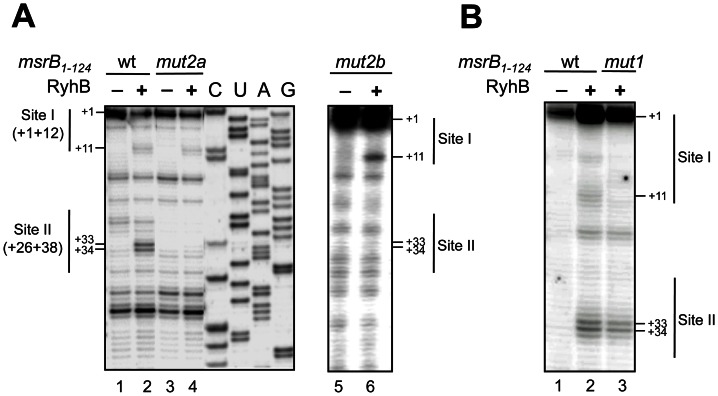
Mutagenesis analysis of the RyhB/*msrB* interaction *in vitro*. (A–B) Autoradiograms of primer extension analysis of *msrB* mRNA are shown (for details, see Materials and Methods, and Results). (A) with wild type (lanes 1–2), mut2a (lanes 3,4) and mut2b (lanes 5,6) *msr*B*_1–124_* transcripts as a template for the reverse transcriptase. Lanes 1,3 and 5: extension with no other component added; lanes 2,4 and 6: extension with RyhB. C, U, A, and G are sequencing lanes obtained using the same radiolabeled primer as in the reverse extension analysis. (B) cDNA extension experiments with wild type (lanes 1–2) and mut1 (lane 3) *msr*B*_1–124_* transcripts as templates for the reverse transcriptase. Lane 1: extension with no other component added; lanes 2–3: extension with RyhB. For (A) and (B), reverse transcriptase stops are indicated at positions +11, +33 and +34. Thin vertical lines indicate nucleotides involved in Site I and Site II. The transcription start of *msrB* is referred to as the position + 1. The numbers to the left indicate sequence positions with respect to the transcription start site.

In order to validate the binding of RyhB at Sites I and II *in vitro*, nucleotide changes were made in the sequences of *msrB* Sites I and II. The consequences on RyhB binding were analysed by PEA. First, mutations (G32C, C33U, A34C) were introduced into the Site II, yielding an *msrB* allele referred to as *mut2a* (Figure 3A). Results showed only one primer extension product, located within the Site I (Figure 5A; lane 4). Thus, modifications introduced into the Site II prevented RyhB to bind at this site. Second, mutations (G6A, U7C, G8A) were introduced into the Site I, yielding the *mut1* allele (Figure 3A). Results showed only one primer extension product, located within Site II (Figure 5B; lane 3). Altogether these mutational analyses further support the notion of an interaction between RyhB and *msrB* at both Sites I and II *in vitro*.

### RyhB binding on the *msrB* mRNA blocks ribosome binding

**Figure 6:**
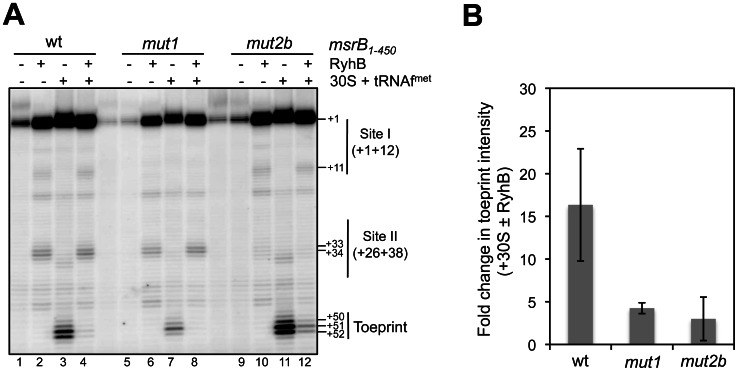
RyhB binding at Site I and Site II blocks ribosome binding to the *msrB* translation initiation region. (A) An autoradiogram of a toeprint analysis is shown (for details, see Materials and Methods, and Results). *msrB_1–450_* wild type and variants (mut1 and mut2b) were used as a template (5 nM) in the cDNA extension experiment. Lanes (1, 5, 9): extension with no other component added; lanes (2, 6, 10): extension with RyhB alone (2.5 μM); in lanes (3, 7, 11): extension with 30S subunit (+initiator tRNA^fmet^) alone (500 nM) ; lanes (4, 8, 12): cDNA extension with 30S subunits (500 nM) along with RyhB (2.5 μM). Thin vertical lines indicate nucleotides involved in Site I and Site II. The transcription start of *msrB* is referred to as the position + 1. The 30S subunit-induced reverse-transcriptase (RT) toeprint is indicated at positions +50 to +52. Other indicated positions are numbered accordingly. (B) Quantification of band intensity was performed by using Image J software and expressed in terms of fold change in toeprint intensity. Fold change represents decrease in toeprint intensity obtained when comparing the two conditions, with 30S ribosomal subunits alone and with 30S ribosomal subunits along with RyhB. Standard error of two independent experiments is shown.

RyhB is known to interfere with translation initiation. Therefore, we asked whether RyhB binding on *msrB* mRNA would block ribosome binding *in vitro*. For this purpose, toeprint assays were run. A long version of *msrB* mRNA (450nt; nt +1 to nt +449) including the whole 5′ UTR region was transcribed *in vitro* and used as a template for toeprint assays (Figure 6). When the 30S ribosomal subunit was added, its binding to the wild type *msrB* mRNA blocked RT elongation, resulting in a shortened cDNA called ‘toeprint’ at position +50 to +52 (Figure 6A–B; lane 3). However, in the presence of RyhB, the intensity of the toeprint band was 16±6 times decreased (Figure 6A–B; compare lane 3 with lane 4). This result shows that the interaction of RyhB with *msrB* mRNA interferes with the binding of the 30S subunit.

We then carried out a toeprint assay with the *msrB mut1* mRNA as a template. No specific primer extension product was observed on the *msrB mut1* mRNA alone (Figure 6A–B; lane 5). In the presence of the 30S ribosomal subunit, a toeprint was detected (Figure 6A–B; lane 7). When RyhB was added, a specific primer extension product was observed at Site II but not at Site I as predicted (Figure 6A–B; lane 6). When both RyhB and the 30S subunit were added, the intensity of the toeprint band was 4.25±0.6 times reduced (Figure 6A–B; compare lane 7 and lane 8). This observation indicates that the interaction of RyhB with Site II efficiently interferes with the 30S binding. However since the interaction of RyhB with Site II does not fully abolish the toeprinting signal it suggests that the pairing of RyhB at Site I may contribute to the inhibitory effect of RyhB on the ribosome binding.

We then tested the effect of mutations in *msrB* RyhB-binding site II. As the *mut2a* mutation used (G32C, C33U, A34C) alters the Shine-Dalgarno sequence, we expected it to reduce the binding affinity of the 30S subunit. A Western-blot analysis of the resulting mutant was carried out and indeed shown to have a very low level of MsrB production (Figure S1). Therefore a 10-fold higher concentration of 30S subunit was used. However, even with such an increased concentration, we failed to observe any detectable toeprint (data not shown). Consequently, we decided to construct a new variant, referred to as *mut2b*. For this, nucleotides C33 and A34 were substituted for A and U, respectively (Figure 3A). A Western-blot analysis of the resulting mutant showed that the MsrB protein was produced at a similar level as the wild type, showing that the mutation had not altered translation efficiency (Figure S1). A primer extension analysis of *mut2b* revealed Site I but not Site II as expected (Figure 5A). Last, we performed a toeprint assay on the *msrB mut2b* mRNA alone and observed no specific primer extension product (Figure 6A–B; lane 9). When RyhB was added, a primer extension product was observed at Site I but no longer at Site II as expected from the mutations introduced (Figure 6A–B; lane 10). Adding the 30S ribosomal subunit significantly increased the toeprint band (Figure 6A–B; lane 11), indicating that the mutation allows efficient ribosome binding. When both RyhB and the 30S subunit were added to the *mut2b* mRNA template, a toeprint band remained visible, although with a 3 times reduced intensity compared to that obtained in the presence of the 30S alone (Figure 6A–B; compare lane 11 with lane 12). These results are consistent with the notion that the binding of RyhB to the Site I could perturb the 30S ribosome binding at the Shine-Dalgarno sequence, although one should be very cautious with such interpretation given the substantial margin of error of the experimental results.

### Disrupting one RyhB binding site is not sufficient to fully restore *msrB* stability upon iron starvation

**Figure 7:**
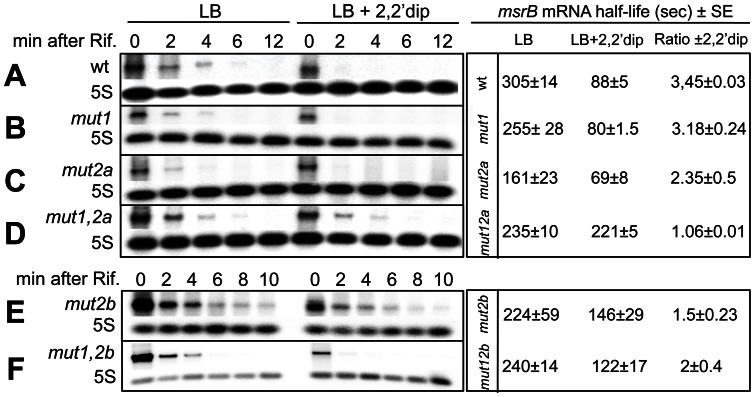
Effect of mutations in RyhB-binding Sites I and II on *msrB* mRNA stability. Northern blot analysis of wild type *msrB* (A), *msrB* mut1 (B), *msrB* mut2a (C), *msrB* mut1,2a (D), *msrB* mut2b (E), and *msrB* mut1,2b (F). Strains were grown at 37°C to an O.D._600_ of 0.4. After 10 min of incubation with 2,2′dip, rifampicin was added. Samples were removed at the times indicated after rifampicin addition and total RNA was extracted as described in Materials and Methods. Half-life of *msrB* mRNA was calculated with or without iron chelator. For determination of *msrB* mRNA amount, 10 μg of total RNA samples was loaded on a denaturating 1.2% agarose gel. After migration, a Northern blot hybridization was performed with a specific oligoprobe for *msrB* and with *5S* as a loading control. Half-life (seconds) of *msrB* mRNA (wild type and mutants) and the ratio of *msrB* mRNA half-life ±2,2′dip, are indicated. Band intensity of *msrB* transcript was normalized to that of 5S RNA.

We investigated the role of each Site I and II in the regulation of *msrB* expression *in vivo*. For this, the half-life of *msrB* wild type and mutant derivative mRNAs, extracted from strains grown in the presence and in the absence of 2,2′dip, was analyzed (Figure 7). The *mut1* half-life was 1.2 times lower than that of wild type in the absence of 2,2′dip (Figure 7B). However, it remained modified, like the wild type, as a function of iron availability (Figure 7B). We found that *mut2a* half-life was reduced as compared with that of the wild type (Figure 7C). Here again, the decay kinetics were faster in the presence of *2,2*′dip (Figure 7C). The rate of *mut2b* decay was similar to that of the wild type (Figure 7E). However, its stability was reduced only 1.5 times under iron limitation, as compared with a 3.5-fold for the wild type (Figure 7E). These results indicate that modifications introduced at Site I or Site II can alter either intrinsic stability (e.g. *mut1* and *mut2a*) or iron-mediated destabilization (e.g. *mut2b*) of the cognate *msrB* mRNAs.

Then, we investigated the effect of combining mutations at both Sites I and II. Unexpectedly the same *mut1* mutation had opposite effects when combined with *mut2a* or *mut2b* mutations. The half-life of *mut1,2a* (Figure 7D) was 1.3 times decreased compared to that of the wild type whereas that of *mut2a* was 1.9 times reduced (Figure 7C). In addition, the stability of *mut1,2a* was not modified as a function of the presence of 2,2′dip (Figure 7D), suggesting that combining the two *mut1* and *mut2a* mutations had an additive effect, which eventually disrupted RyhB control. Surprisingly, combining *mut1* and *mut2b* yielded an mRNA species much less stable than the *mut2b* allele. In comparison to *mut2a*, the stability of *mut2b* was less altered by iron limitation. This last result contrasted with the behavior of the *mut2b* species, which had been found marginally influenced by iron availability (see Figure 7E). These results indicated that the *mut1* mutation has apparently suppressed the effect of the *mut2b* mutation (Figure 7F). Altogether, this *in vivo* analysis demonstrates that introducing modifications at Sites I or II can have an effect on *msrB* mRNA half-life and possibly its control by iron availability.

### *msrA* gene expression is unaffected when iron becomes scarce

Transcriptional and translational fusions between the *msrA* gene and the *lacZ* reporter gene were constructed and inserted at the *att* site in the *E. coli* chromosome. The engineered strains namely JB55 and JB56, respectively, were grown in LB medium supplemented or not with 2,2′dip. Expression of both the transcriptional and the translational fusion were 1.4-fold increased under iron deprivation (Table 3). However, introduction of a *ryhB*, a *fur* or a *ryhB fur* mutations did not change significantly *msrA’-‘lacZ* expression levels (Table 3). These results suggested that unlike *msrB*, *msrA* gene expression might be induced by iron availability but not by a Fur or RyhB dependent mecanism.

## Discussion

This study demonstrates that the synthesis of the methionine sulfoxide reductase MsrB is repressed under iron limitation. We show this negative regulation to result from the action of the sRNA RyhB. The involvement of additional *trans*-acting factors, such as the transcriptional iron sensing repressor Fur, the RNA chaperone Hfq and the degradosome-belonging RNase E was also established [43], [44], [45], [46]. The *msrB* gene is therefore a new member of the RyhB regulon, as initially suggested by transcriptomic analysis of the Massé group [30]. The RyhB-mediated down regulation of *msrB* is likely to be related to the fact that MsrB is an iron-binding enzyme [38].

The mechanism allowing RyhB to control expression of the *msrB* gene appears to be original and complex. Indeed, the *msrB* gene was found to contain two binding sites for RyhB. The upstream site is located at the very 5′ end of the *msrB* mRNA while the second site is located 30 nucleotides downstream and overlaps the Shine-Dalgarno sequence. Previous studies on RyhB-mediated regulation of the *sodB*, *sdhD* or *iscS* genes showed that the RyhB-binding site overlaps the Shine-Dalgarno sequence, thereby inhibiting translation initiation and provoking mRNA instability [31], [35]. The region of RyhB (loop II) that is used to target *msrB* is the same as that pairing with the 5′ UTR regions of *sodB* or *sdhD*. Use of molecular techniques allowed us to demonstrate RyhB binding at both sites *in vitro*. In Salmonella, the RybB sRNA has multiple binding sites within the *ompCD* mRNA but RybB binds only one site targeting either *ompC* or *ompD* mRNA as the pairing sites are mutually exclusive [47]. Also, a recent study has reported that SgrS sRNA can have multiple binding sites within a polycistronic mRNAs [48]. However, the binding sites of SgrS are present within portions of the mRNA that are encoded by different genes of the operon. Hence, all of these cases differ from that described here where the same sRNA can bind to two different locations within the same mRNA species. Curiously, the two sites show the same 9 nucleotide sequence. Evidently, this raises the question of whether two RyhB molecules bind simultaneously to *msrB* mRNA or only one at a time at one of the two sites. Further experiments aimed at establishing the stoichiometry *msrB*/RyhB will have to be carried out to get insight into this question.

Toeprint analysis revealed that the pairing of RyhB with the *msrB* mRNA at Site II prevents the binding of the 30S ribosomal subunit while it remains unclear if pairing at Site I bears any effect on the binding of the ribosome. The way Site II-bound RyhB interferes with the ribosome binding is likely to be due to steric hindrance as Site II overlaps the Shine-Dalgarno sequence. Such a direct competition between RyhB and the ribosome was demonstrated for the expression of *sodB*, *sdh* and *iscS* genes. In contrast, the way Site I-bound RyhB may interfere with ribosome binding, if it does, is uncertain. In *Salmonella typhimurium*, the regulatory sRNA GcvB represses translation by binding to target RNA *gltI* and *argT* at upstream sites, outside the RBS [49]. In Salmonella, RybB targets sites on *omp* mRNAs within the coding region [50] or upstream of the region covered by the ribosome [51]. One possibility is that it might directly interfere with the binding of the ribosome. Indeed the ribosome was proposed to cover a region extending from – 35 nt upstream the start codon to + 19 nt downstream [52]. An alternative role of the pairing of RyhB at Site I might be that it stimulates the degradation of *msrB* mRNA by RNase E and the degradosome as it has recently been reported for *sodB* mRNA by the Massé group [37]. In-depth study of the decay of *mut1* mRNA species is required to assess the possibility of such a mechanism.

Analysis of *msrB* alleles that contained modified Site I and/or Site II highlighted the complexity of the mechanism underlying RyhB regulation, yet provided us with important information. In particular, we were able to isolate mutations within the regulatory sequence of *msrB* transcript that uncoupled *msrB* degradation and iron bioavailability. However, these mutants exhibited additional unexpected features. One case is the “double” *mut1,2a* allele, which contains a mutation at each Site I and II. This allele exhibited a constitutive-like behaviour as the half-life of the cognate mRNA, was not altered in bacteria that are grown under iron limitation. This departs from the behaviours of both “single” *mut1* and *mut2a* alleles, which remain regulated by iron and do not show any increased stability. Thus, the additive effect of the two mutations could signify the involvement of the two sites in RyhB-mediated regulation. However, *mut1,2a* allele is very poorly recognized by the 30S ribosomal subunit and, accordingly, it is very poorly translated. Hence, it is impossible to decipher whether the loss of the iron regulation is due to the inability of RyhB to recognize this species and to interfere with ribosome binding or if reduced ribosome binding efficiency prevents any associated regulatory mechanism to take place. Moreover, one would have expected the half-life of this mutated version to be drastically shortened, as it is no longer protected by ribosomes. Could the two mutations yield a highly structured mRNA, unable to interact with any of the *trans*-acting factors, including the ribosome, RyhB, the degradosome or a subset of them? A thorough RNase structural probing is needed prior to further interpret the behaviour of this species. Another puzzling case is the *mut2b* allele. In this case, the sole modification of the Site II was sufficient to alter iron regulation. *In vitro* analysis of the *mut2b* mRNA species revealed that RyhB no longer interacts with Site II but interacts with Site I. The iron regulation was altered but not fully abolished and this was consistent with the notion that the RyhB/Site I interaction can mediate iron regulation. However, a simple interpretation of this allele was prevented by the observation that the *mut2b* mutation effect could be moderately suppressed by the *mut1* mutation. Indeed, the *mut1,2b* allele produces an mRNA species which remains under the control of iron bioavailibility with a 2-fold decrease in its stability upon 2,2′ dip treatment. Clearly more in-depth analysis of the cognate structure is required before we make any firm conclusion on how they affect the *msrB*/RyhB interaction, and the regulation of the *msrB* expression.

Methionine sulfoxide reductases MsrA and MsrB catalyse the reduction of S- and R-diastereoisomeric forms of methionine sulfoxide. Previous studies established that the combined action of both MsrA and MsrB is required for full repair of oxidized polypeptides [8], [54]. From this biochemical point of view, one would expect that the two enzymes will be synthesized in the same conditions. This is likely to be the case in *Bacillus subtilis* wherein the two cognate structural genes belong to the same operon [55]. This is also the case of *Neisseria* wherein both MsrA and MsrB are actually fused into a single polypeptide [27]. Conversely, in *E. coli*, the two genes are physically separated, raising the question of their co-regulation. Further, we found that MsrB synthesis is decreased under iron limitation conditions whereas that of MsrA appears essentially not affected by this limitation. One might speculate that under iron limitation, damage by iron-mediated Fenton reaction is reduced and methionine oxidation is infrequent. This might explain why *E. coli* reduces MsrB synthesis while saving iron for essential processes and relies only on MsrA to rescue those few oxidized polypeptides.

## Supporting Information

Figure S1 **Western blot analysis of MsrB protein levels from cells expressing wild type** ***msrB*** **and mutant** ***msrB***. Wild type *msrB* (A) and mutant *msrB* (mut2a (B) and mut2b (C)). Strains were grown at 37°C to an O.D._600_ of 0.4. After 60 min of incubation with 2,2’dip, samples were removed and proteins were extracted as described in Materials and Methods. MsrB proteins were probed using anti-HA antibodies.
(TIF)

## References

[1] Finkel T, Holbrook NJ (2000) Oxidants, oxidative stress and the biology of ageing. Nature 408: 239–247. 11089981 10.1038/35041687

[2] Brosnan JT, Brosnan ME (2006) The sulfur-containing amino acids: an overview. J Nutr 136: 1636S–1640S. 16702333 10.1093/jn/136.6.1636S

[3] Maisonneuve E, Ducret A, Khoueiry P, Lignon S, Longhi S, et al (2009) Rules governing selective protein carbonylation. PLoS One 4: e7269. 19802390 10.1371/journal.pone.0007269 PMC2751825

[4] Maisonneuve E, Ezraty B, Dukan S (2008) Protein aggregates: an aging factor involved in cell death. J Bacteriol 190: 6070–6075. 18621895 10.1128/JB.00736-08 PMC2546795

[5] Moskovitz J, Oien DB (2010) Protein carbonyl and the methionine sulfoxide reductase system. Antioxid Redox Signal 12: 405–415. 19686038 10.1089/ars.2009.2809

[6] Boschi-Muller S, Olry A, Antoine M, Branlant G (2005) The enzymology and biochemistry of methionine sulfoxide reductases. Biochim Biophys Acta 1703: 231–238. 15680231 10.1016/j.bbapap.2004.09.016

[7] Ezraty B, Aussel L, Barras F (2005) Methionine sulfoxide reductases in prokaryotes. Biochim Biophys Acta 1703: 221–229. 15680230 10.1016/j.bbapap.2004.08.017

[8] Grimaud R, Ezraty B, Mitchell JK, Lafitte D, Briand C, et al (2001) Repair of oxidized proteins. Identification of a new methionine sulfoxide reductase. J Biol Chem 276: 48915–48920. 11677230 10.1074/jbc.M105509200

[9] Sharov VS, Ferrington DA, Squier TC, Schoneich C (1999) Diastereoselective reduction of protein-bound methionine sulfoxide by methionine sulfoxide reductase. FEBS Lett 455: 247–250. 10437782 10.1016/s0014-5793(99)00888-1

[10] Sharov VS, Schoneich C (2000) Diastereoselective protein methionine oxidation by reactive oxygen species and diastereoselective repair by methionine sulfoxide reductase. Free Radic Biol Med 29: 986–994. 11084287 10.1016/s0891-5849(00)00400-7

[11] Luo S, Levine RL (2009) Methionine in proteins defends against oxidative stress. FASEB J 23: 464–472. 18845767 10.1096/fj.08-118414 PMC2630790

[12] Koc A, Gladyshev VN (2007) Methionine sulfoxide reduction and the aging process. Ann N Y Acad Sci 1100: 383–386. 17460202 10.1196/annals.1395.042

[13] Zhang XH, Weissbach H (2008) Origin and evolution of the protein-repairing enzymes methionine sulphoxide reductases. Biol Rev Camb Philos Soc 83: 249–257. 18557976 10.1111/j.1469-185X.2008.00042.x

[14] Boschi-Muller S, Gand A, Branlant G (2008) The methionine sulfoxide reductases: Catalysis and substrate specificities. Arch Biochem Biophys 474: 266–273. 18302927 10.1016/j.abb.2008.02.007

[15] Hansel A, Heinemann SH, Hoshi T (2005) Heterogeneity and function of mammalian MSRs: enzymes for repair, protection and regulation. Biochim Biophys Acta 1703: 239–247. 15680232 10.1016/j.bbapap.2004.09.010

[16] Hansel A, Jung S, Hoshi T, Heinemann SH (2003) A second human methionine sulfoxide reductase (hMSRB2) reducing methionine-R-sulfoxide displays a tissue expression pattern distinct from hMSRB1. Redox Rep 8: 384–388. 14980072 10.1179/135100003225003429

[17] Moskovitz J (2005) Roles of methionine suldfoxide reductases in antioxidant defense, protein regulation and survival. Curr Pharm Des 11: 1451–1457. 15853675 10.2174/1381612053507846

[18] Ruan H, Tang XD, Chen ML, Joiner ML, Sun G, et al (2002) High-quality life extension by the enzyme peptide methionine sulfoxide reductase. Proc Natl Acad Sci U S A 99: 2748–2753. 11867705 10.1073/pnas.032671199 PMC122419

[19] Moskovitz J, Berlett BS, Poston JM, Stadtman ER (1997) The yeast peptide-methionine sulfoxide reductase functions as an antioxidant in vivo. Proc Natl Acad Sci U S A 94: 9585–9589. 9275166 10.1073/pnas.94.18.9585 PMC23225

[20] Moskovitz J, Bar-Noy S, Williams WM, Requena J, Berlett BS, et al (2001) Methionine sulfoxide reductase (MsrA) is a regulator of antioxidant defense and lifespan in mammals. Proc Natl Acad Sci U S A 98: 12920–12925. 11606777 10.1073/pnas.231472998 PMC60800

[21] Rouhier N, Vieira Dos Santos C, Tarrago L, Rey P (2006) Plant methionine sulfoxide reductase A and B multigenic families. Photosynth Res 89: 247–262. 17031545 10.1007/s11120-006-9097-1

[22] Ezraty B, Bos J, Barras F, Aussel L (2005) Methionine sulfoxide reduction and assimilation in Escherichia coli: new role for the biotin sulfoxide reductase BisC. J Bacteriol 187: 231–237. 15601707 10.1128/JB.187.1.231-237.2005 PMC538846

[23] Douglas T, Daniel DS, Parida BK, Jagannath C, Dhandayuthapani S (2004) Methionine sulfoxide reductase A (MsrA) deficiency affects the survival of Mycobacterium smegmatis within macrophages. J Bacteriol 186: 3590–3598. 15150247 10.1128/JB.186.11.3590-3598.2004 PMC415777

[24] Singh VK, Moskovitz J (2003) Multiple methionine sulfoxide reductase genes in Staphylococcus aureus: expression of activity and roles in tolerance of oxidative stress. Microbiology 149: 2739–2747. 14523107 10.1099/mic.0.26442-0

[25] Hassouni ME, Chambost JP, Expert D, Van Gijsegem F, Barras F (1999) The minimal gene set member msrA, encoding peptide methionine sulfoxide reductase, is a virulence determinant of the plant pathogen Erwinia chrysanthemi. Proc Natl Acad Sci U S A 96: 887–892. 9927663 10.1073/pnas.96.3.887 PMC15320

[26] Denkel LA, Horst SA, Rouf SF, Kitowski V, Bohm OM, et al (2011) Methionine sulfoxide reductases are essential for virulence of Salmonella typhimurium. PLoS One 6: e26974. 22073230 10.1371/journal.pone.0026974 PMC3206869

[27] Skaar EP, Tobiason DM, Quick J, Judd RC, Weissbach H, et al (2002) The outer membrane localization of the Neisseria gonorrhoeae MsrA/B is involved in survival against reactive oxygen species. Proc Natl Acad Sci U S A 99: 10108–10113. 12096194 10.1073/pnas.152334799 PMC126632

[28] Dhandayuthapani S, Blaylock MW, Bebear CM, Rasmussen WG, Baseman JB (2001) Peptide methionine sulfoxide reductase (MsrA) is a virulence determinant in Mycoplasma genitalium. J Bacteriol 183: 5645–5650. 11544227 10.1128/JB.183.19.5645-5650.2001 PMC95456

[29] Wizemann TM, Moskovitz J, Pearce BJ, Cundell D, Arvidson CG, et al (1996) Peptide methionine sulfoxide reductase contributes to the maintenance of adhesins in three major pathogens. Proc Natl Acad Sci U S A 93: 7985–7990. 8755589 10.1073/pnas.93.15.7985 PMC38861

[30] Masse E, Vanderpool CK, Gottesman S (2005) Effect of RyhB small RNA on global iron use in Escherichia coli. J Bacteriol 187: 6962–6971. 16199566 10.1128/JB.187.20.6962-6971.2005 PMC1251601

[31] Masse E, Gottesman S (2002) A small RNA regulates the expression of genes involved in iron metabolism in Escherichia coli. Proc Natl Acad Sci U S A 99: 4620–4625. 11917098 10.1073/pnas.032066599 PMC123697

[32] Masse E, Salvail H, Desnoyers G, Arguin M (2007) Small RNAs controlling iron metabolism. Curr Opin Microbiol 10: 140–145. 17383226 10.1016/j.mib.2007.03.013

[33] Masse E, Escorcia FE, Gottesman S (2003) Coupled degradation of a small regulatory RNA and its mRNA targets in Escherichia coli. Genes Dev 17: 2374–2383. 12975324 10.1101/gad.1127103 PMC218075

[34] Cornelis P, Wei Q, Andrews SC, Vinckx T (2011) Iron homeostasis and management of oxidative stress response in bacteria. Metallomics 3: 540–549. 21566833 10.1039/c1mt00022e

[35] Desnoyers G, Morissette A, Prevost K, Masse E (2009) Small RNA-induced differential degradation of the polycistronic mRNA iscRSUA. EMBO J 28: 1551–1561. 19407815 10.1038/emboj.2009.116 PMC2693151

[36] Prevost K, Salvail H, Desnoyers G, Jacques JF, Phaneuf E, et al (2007) The small RNA RyhB activates the translation of shiA mRNA encoding a permease of shikimate, a compound involved in siderophore synthesis. Mol Microbiol 64: 1260–1273. 17542919 10.1111/j.1365-2958.2007.05733.x

[37] Prevost K, Desnoyers G, Jacques JF, Lavoie F, Masse E (2011) Small RNA-induced mRNA degradation achieved through both translation block and activated cleavage. Genes Dev 25: 385–396. 21289064 10.1101/gad.2001711 PMC3042161

[38] Olry A, Boschi-Muller S, Yu H, Burnel D, Branlant G (2005) Insights into the role of the metal binding site in methionine-R-sulfoxide reductases B. Protein Sci. 14: 2828–2837. 10.1110/ps.051711105 PMC2253221 16251365

[39] Simons RW, Houman F, Kleckner N (1987) Improved single and multicopy lac-based cloning vectors for protein and operon fusions. Gene 53: 85–96. 3596251 10.1016/0378-1119(87)90095-3

[40] Powell BS, Rivas MP, Court DL, Nakamura Y, Turnbough CL Jr (1994) Rapid confirmation of single copy lambda prophage integration by PCR. Nucleic Acids Res 22: 5765–5766. 7838735 10.1093/nar/22.25.5765 PMC310146

[41] Makhno VI, Peshin NN, Semenkov Iu P, Kirillov SV (1988) [A modified method of isolation of "tight" 70S ribosomes from Escherichia coli highly active at different stages of the elongation cycle]. Mol Biol (Mosk) 22: 670–679. 3054495

[42] Haentjens-Sitri J, Allemand F, Springer M, Chiaruttini C (2008) A competition mechanism regulates the translation of the Escherichia coli operon encoding ribosomal proteins L35 and L20. J Mol Biol 375: 612–625. 18037435 10.1016/j.jmb.2007.10.058

[43] Afonyushkin T, Vecerek B, Moll I, Blasi U, Kaberdin VR (2005) Both RNase E and RNase III control the stability of sodB mRNA upon translational inhibition by the small regulatory RNA RyhB. Nucleic Acids Res 33: 1678–1689. 15781494 10.1093/nar/gki313 PMC1069011

[44] Morita T, Maki K, Aiba H (2005) RNase E-based ribonucleoprotein complexes: mechanical basis of mRNA destabilization mediated by bacterial noncoding RNAs. Genes Dev 19: 2176–2186. 16166379 10.1101/gad.1330405 PMC1221888

[45] Geissmann TA, Touati D (2004) Hfq, a new chaperoning role: binding to messenger RNA determines access for small RNA regulator. EMBO J 23: 396–405. 14739933 10.1038/sj.emboj.7600058 PMC1271764

[46] Moll I, Afonyushkin T, Vytvytska O, Kaberdin VR, Blasi U (2003) Coincident Hfq binding and RNase E cleavage sites on mRNA and small regulatory RNAs. RNA 9: 1308–1314. 14561880 10.1261/rna.5850703 PMC1287052

[47] Balbontin R, Fiorini F, Figueroa-Bossi N, Casadesus J, Bossi L (2010) Recognition of heptameric seed sequence underlies multi-target regulation by RybB small RNA in Salmonella enterica. Mol Microbiol 78: 380–394. 20979336 10.1111/j.1365-2958.2010.07342.x

[48] Rice JB, Balasubramanian D, Vanderpool CK (2012) Small RNA binding-site multiplicity involved in translational regulation of a polycistronic mRNA. Proc Natl Acad Sci U S A 109: E2691–2698. 22988087 10.1073/pnas.1207927109 PMC3479541

[49] Sharma CM, Darfeuille F, Plantinga TH, Vogel J (2007) A small RNA regulates multiple ABC transporter mRNAs by targeting C/A-rich elements inside and upstream of ribosome-binding sites. Genes Dev 21: 2804–2817. 17974919 10.1101/gad.447207 PMC2045133

[50] Bouvier M, Sharma CM, Mika F, Nierhaus KH, Vogel J (2008) Small RNA binding to 5′ mRNA coding region inhibits translational initiation. Mol Cell 32: 827–837. 19111662 10.1016/j.molcel.2008.10.027

[51] Papenfort K, Bouvier M, Mika F, Sharma CM, Vogel J (2010) Evidence for an autonomous 5′ target recognition domain in an Hfq-associated small RNA. Proc Natl Acad Sci U S A 107: 20435–20440. 21059903 10.1073/pnas.1009784107 PMC2996696

[52] Huttenhofer A, Noller HF (1994) Footprinting mRNA-ribosome complexes with chemical probes. EMBO J 13: 3892–3901. 8070416 10.1002/j.1460-2075.1994.tb06700.x PMC395302

[53] Zuker M (2003) Mfold web server for nucleic acid folding and hybridization prediction. Nucleic Acids Res 31: 3406–3415. 12824337 10.1093/nar/gkg595 PMC169194

[54] Ezraty B, Grimaud R, El Hassouni M, Moinier D, Barras F (2004) Methionine sulfoxide reductases protect Ffh from oxidative damages in Escherichia coli. EMBO J 23: 1868–1877. 15057280 10.1038/sj.emboj.7600172 PMC394232

[55] You C, Sekowska A, Francetic O, Martin-Verstraete I, Wang Y, et al (2008) Spx mediates oxidative stress regulation of the methionine sulfoxide reductases operon in Bacillus subtilis. BMC Microbiol 8: 128. 18662407 10.1186/1471-2180-8-128 PMC2518928

[56] Plumbridge J, Soll D (1989) Characterization of cis-acting mutations which increase expression of a glnS-lacZ fusion in Escherichia coli. Mol Gen Genet 216: 113–119. 2471922 10.1007/BF00332238

[57] Morita T, Kawamoto H, Mizota T, Inada T, Aiba H (2004) Enolase in the RNA degradosome plays a crucial role in the rapid decay of glucose transporter mRNA in the response to phosphosugar stress in Escherichia coli. Mol Microbiol 54: 1063–1075. 15522087 10.1111/j.1365-2958.2004.04329.x

