# Supplementary figures and images for: The sRNA RyhB Regulates the Synthesis of the Escherichia coli Methionine Sulfoxide Reductase MsrB but Not MsrA
Source: PLoS One. 2013 May 9;8(5):e63647. doi: 10.1371/journal.pone.0063647 (PMC3650055; doi:10.1371/journal.pone.0063647)

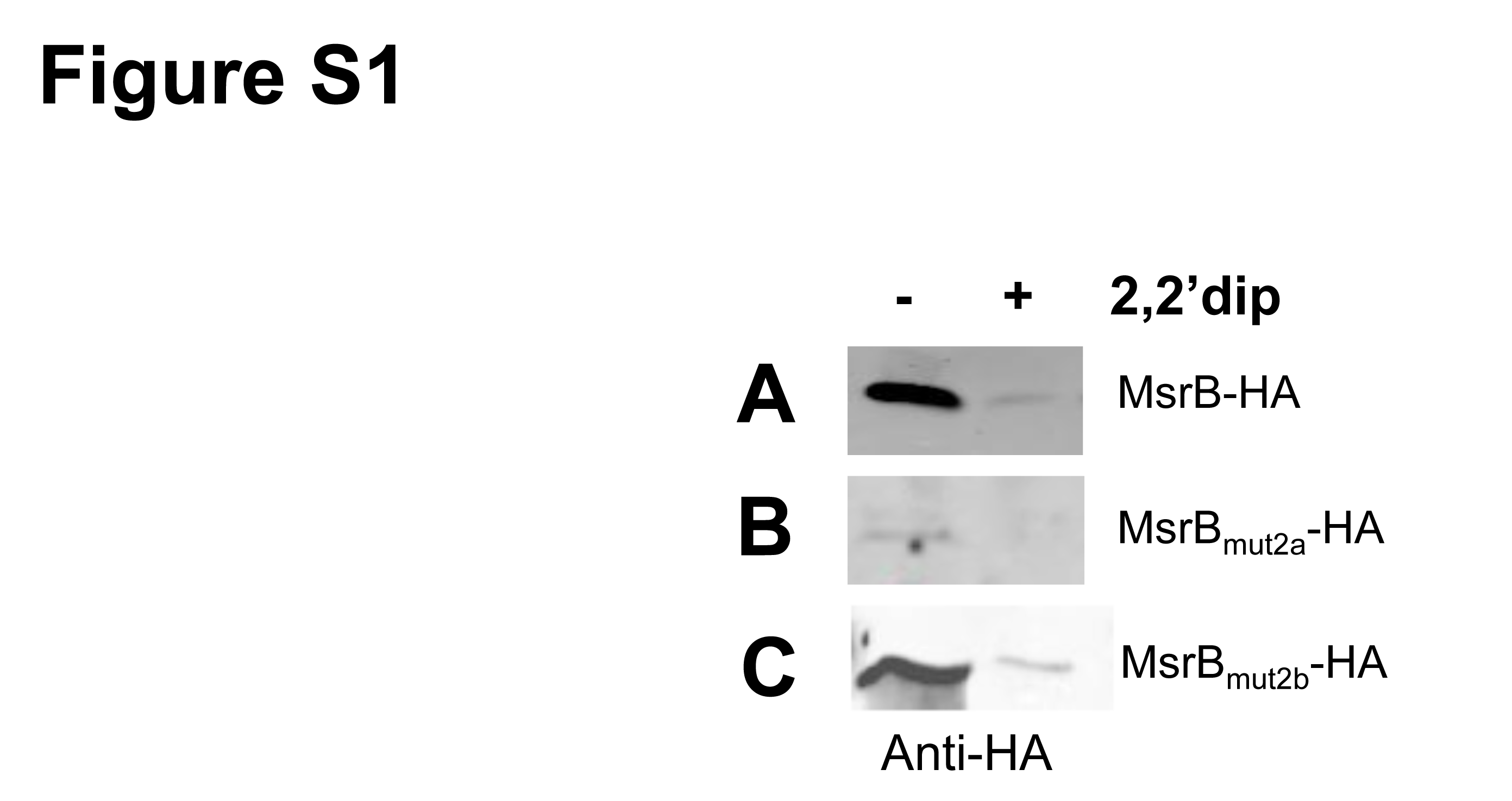

Supplement: Figure S1 — Western blot analysis of MsrB protein levels from cells expressing wild type msrB and mutant msrB. Wild type msrB (A) and mutant msrB (mut2a (B) and mut2b (C)). Strains were grown at 37°C to an O.D.600 of 0.4. After 60 min of incubation with 2,2’dip, samples were removed and proteins were extracted as described in Materials and Methods. MsrB proteins were probed using anti-HA antibodies. (TIF) [file pone.0063647.s001.tif]
